# Association between Serum C-Peptide as a Risk Factor for Cardiovascular Disease and High-Density Lipoprotein Cholesterol Levels in Nondiabetic Individuals

**DOI:** 10.1371/journal.pone.0112281

**Published:** 2015-01-05

**Authors:** Ying Li, Yue Li, Lu Meng, LianShun Zheng

**Affiliations:** 1 Department of Social Medicine, School of Public Health, Zhejiang University, Zhejiang, China; 2 School of Basic Medical Sciences, Zhejiang University, Zhejiang, China; University Heart Center Freiburg, Germany

## Abstract

**Objective:**

Objective: Although serum C-peptide has increasingly received attention as a new and important risk factor for cardiovascular disease (CVD), the potential mechanisms remain unclear. This study aimed to investigate the association between serum C-peptide as a risk factor for CVD and high-density lipoprotein cholesterol (HDL-C) levels.

**Methods:**

The present study included 13,185 participants aged ≥20 years. Serum C-peptide and HDL-C levels were measured according to a standard protocol. Stratified analysis of covariance was used to compare serum HDL-C levels between different quartiles of serum C-peptide levels. Logistic regression analysis was used to determine the association between serum C-peptide and HDL-C levels. Cox proportional hazard regression analysis was conducted to determine the hazard ratio of serum HDL-C for CVD-related mortality.

**Results:**

The results of the ANCOVA analysis showed a significant linear trend between the mean serum HDL-C level and the different quartiles of serum C-peptide. Compared to the first quartile (25th percentile), the second, third, and fourth quartiles had gradual reduction in serum HDL-C levels. Logistic regression analyses showed a strong negative association between serum C-peptide levels and HDL-C levels; the p value for the linear trend was <0.001. In men, compared with the lowest quartile of the serum C-peptide level, the relative risk was 1.75, 2.79, and 3.07 for the upper three quartiles of the serum C-peptide level. The relative risk was 1.60, 2.61, and 3.67 for women. The results of the survival analysis showed that serum HDL-C levels were negatively associated with CVD-related death in both men and women.

**Conclusion:**

Serum C-peptide as a risk factor for CVD was significantly and negatively associated with serum HDL-C levels in individuals without diabetes. These findings suggest that serum C-peptide levels association with CVD death can be caused, at least in part, by the low serum HDL-C level.

## Introduction

Cardiovascular disease (CVD) is the most prevalent disease and leading cause of mortality in the worldwide. Despite a reported 60% decrease in the age-adjusted CVD mortality rate over the past 30 years, the global disease prevalence remains largely unchanged owing to the rapidly aging population [1], [2]. Previous epidemiological studies have identified several traditional risk factors that confer a high probability of future CVD events [3], [4]. In additional, C-reactive protein and homocysteine, as conditional risk factors, are reportedly associated with an increased risk of CVD [5]. Recent studies have also reported serum C-peptide as a new and important risk factor for CVD-related or overall death in nondiabetic adults [6].

Serum C-peptide, which was long considered biologically inactive, is known as an indicator of insulin resistance and a useful marker of beta-cell function [7]. However, a growing number of recent studies have shown that it is an active peptide with important physiologic functions [8]. In several studies, serum C-peptide was shown to affect microvascular blood flow and to improve nerve or renal function in animal models and in humans with type 1 diabetes [9]–[12]. Other studies suggested that serum C-peptide may be deposited in the vessel wall during early atherogenesis and promote the recruitment of monocytes and CD4-positive lymphocytes via chemotactic effects in some individuals with diabetes [13], [14]. Although it has been established that serum C-peptide is a marker of insulin resistance and obesity, specifically in type 2 diabetes, and basal C-peptide levels are significantly elevated in patients with metabolic syndrome and diabetes [15], more recent studies have reported that serum C-peptide levels are strongly and positively associated with stroke events independent of serum insulin level in people with diabetes [16]. A nationwide study suggested that serum C-peptide levels are a better predictor of cardiovascular-related and total mortality than serum insulin and other derived measures of insulin resistance in nondiabetic individuals; furthermore, the effects are independent of several major risk factors for the development of diabetes or CVD [17]. The complex mechanisms underlying the association between serum C-peptide levels and CVD-related death remain unclear. Several other studies have also reported an increased risk of breast cancer with higher C-peptide levels in nondiabetic women. Increasing attention is being paid to the potential mechanisms for serum C-peptide as an independent risk factor for CVD-related mortality in nondiabetic individuals.

It has long been known that elevated low-density lipoprotein cholesterol (LDL-C) levels are among the major risk factors for CVD events in both men and women [18], [19]. Conversely, high serum high-density lipoprotein cholesterol (HDL-C) levels are associated with a reduced risk for CVD through reduced oxidation, vascular thrombosis, and inflammation in addition to improved endothelial function and promotion of endothelial repair [20]. Recently, a number of studies have suggested that low HDL-C levels are an independent risk factor for CVD events and should be considered as a new treatment target. In the United States, it is estimated that approximately 35% of men and 39% of women have low serum HDL-C levels [21]. Therefore, this important and widely prevalent risk factor should be addressed in the clinical context of primordial or primary prevention. HDL-C deficiency may result from several major causes, including smoking, abdominal obesity, and type 2 diabetes to primary or familial causes, such as specific genetic mutations [22]. However, the etiology of low HDL-C levels remains unclear.

We hypothesized that serum C-peptide is an important factor resulting in reduced HDL-C levels, and low levels of HDL-C may lead to CVD-related mortality in individuals without diabetes. To test this hypothesis, we compared HDL-C levels based on different quartiles of serum C-peptide levels using data from a nationally representative sample of the general population without diabetes and evaluated the risk of lower HDL-C levels caused by different levels of serum C-peptide. Additionally, we verified the association between HDL-C levels and CVD-related death, independent of other known risk factors.

## Materials and Methods

### Subjects

This study was based on data from the National Health and Nutrition Examination Survey III (NHANES III), a population-based survey that aimed to assess the health and nutritional status in the general population of the United States. Baseline data were collected from 1988 to 1994 using a complex, multistage cluster sampling design. A representative sample comprising a total of 33,994 US residents aged ≥2 months was identified. The mortality follow-up was conducted by linking the records to National Death Index records. Details about the original survey and its methods have been described elsewhere [23]. All participants provided written informed consent, if the participant is 17 years or younger, the written informed consent will also be obtained from parental. The study was approved by the institutional review board at the Centers for Disease Control and Prevention (Atlanta, Georgia).

### Baseline information collection

The NHANES III survey consisted of 2 parts: a home interview and a health examination. In the home interview, the participants self-reported their age, race/ethnicity, general health status, diet, and physical activity level; medical history was obtained during the household interview. With regard to physical activity, the participants were asked whether they performed specific leisure time exercise or physical activities in the past month, and the frequency of reported exercise, sports, or physically active hobbies was also recorded. Information regarding tobacco use was collected for the age of initiation, frequency, duration, and amount smoked. Smoking status was classified as never, former, or current smoker. Drinkers were identified as respondents who reported that they had consumed at least 12 drinks during the past 12 months. The participants reported the highest grade or year of regular school. The health examination was carried out in a large mobile examination center (MEC).

### Biochemical measurements

Serum C-peptide (nmol/L) was measured using the radioimmunoassay method. Blood samples from fasting subjects were collected for C-peptide analysis in accordance with the NHANES sample collection criteria. Venous blood (3–5 mL) was drawn in vacuum tubes. Specimens were allowed to clot at 20–25°C for 15–30 min and were then centrifuged at 1500× *g* for 10 min. Serum was transferred to a 2-mL polypropylene screw-top vial and frozen at −70°C until analysis. The analytical assay was determined to have a precision of ≤10% of the total coefficient of variation.

Serum HDL-C (mmol/L) was measured following the precipitation of the other lipoproteins with a polyanion/divalent cation mixture. Then, 100 µL of heparin sulfate-MnCl mixture was added to 1 mL serum for each sample. The precipitate was removed by centrifugation at 1500× *g* for 30 min. The clear supernatant was removed and placed in a 20 mL glass vial. Then, 500 µL of the supernatant and 50 µL sodium bicarbonate were placed in an Eppendorf tube and vortexed intermittently. The tubes were left to stand at 20–25°C for 10 min; they were then centrifuged at 10,000× *g* for 2 min. The HDL-C in the clear supernatant was measured. A detailed description of the quality assurance and quality control procedures can be found in the laboratory procedures used for the NHANES III [24].

### Anthropometrics and blood pressure measurements

Weight, standing height, and waist circumference (WC) were measured according to a standard protocol at the MEC. Height was measured using an electronic stadiometer, and weight was measured using a digital scale connected to the integrated survey information system. Body mass index (BMI) was calculated as the weight in kilograms divided by the square of the height in meters (kg/m^2^). Blood pressure (BP) measurements were performed on two occasions, and three BP readings were obtained for each occasion. The first occasion was in the home by an interviewer and the second by a physician at the MEC. We used the BP measurement data from the MEC in the present study.

### Outcome measurements

The mortality status of the participants in the NHANES III was ascertained in December 31, 2006 using the National Death Index. In this study, major CVD mortality included deaths coded for hypertensive disease (International Classification of Diseases, 10th Revision [ICD-10] codes I00–I78).

### Statistical analysis

We restricted our analysis to adults aged ≥20 years for whom serum C-peptide and HDL-C measurements were available. We excluded participants with self-reported diabetes, a fasting plasma glucose (FPG) level ≥126 mg/dL, or glycohemoglobin level ≥6.5%. In addition, participants were also excluded if they were taking lipid-lowering drugs. As a result, the analysis included 13,185 subjects (6,228 men and 6,957 women). Descriptive statistics were conducted for the baseline characteristics of the study participants. Sex-specific categorical variables were presented as weighted percentages and continuous variables as the mean and standard deviation. We used the final MEC examination weight for the analysis; weighted percentages were calculated to describe the baseline characteristics.

We conducted stratified analysis of covariance (ANCOVA) to evaluate the change in serum HDL-C levels based on the quartiles of the serum C-peptide levels. The stratification factors were decided as follows: sex (male or female); BMI (<25 or ≥25 kg/m^2^), and serum insulin level (<36.78, 36.78–74.95 or >74.95 pmol/L). We performed a variance homogeneity test and a normality test. In each separate ANCOVA model by sex, BMI, and serum insulin level, the serum HDL-C level was the dependent variable, and the independent variable was the quartiles of serum C-peptide levels. The serum C-peptide levels were divided into four categories based on the quartiles. The first quartile was defined as the 25th percentile (0.365 nmol/L), the median value represented the 50th percentile (0.605 nmol/L), and the third quartile was defined as the 75th percentile (0.929 nmol/L). The comparisons were made between the four categories using an F test with a significance level of 0.05, and the linear trend was assessed using a general linear model; the first quartile was used as the reference group. The mean and standard error were calculated for serum HDL-C levels according to the quartiles of serum C-peptide levels for each stratification factors. The ANCOVA covariates included age, race-ethnicity, education levels, physical activity, smoking status, and alcohol use.

Logistic regression analysis was used to evaluate the odds ratios (ORs) for the association between serum C-peptide levels and serum HDL-C levels. Serum HDL-C was treated as a binary variable. The serum C-peptide levels were divided into quartiles; three dummy variables were defined, with the lowest quartile as the reference group. The model was adjusted for age, race-ethnicity, education levels, physical activity, smoking status, alcohol use, systolic blood pressure, serum insulin level, serum triglyceride level, and serum cholesterol level.

Cox proportional hazards regression analysis was used to identify the association between serum HDL-C levels and CVD-related death. Three dummy variables were defined using the quartiles; the cut off value was determined for the 25th (1.03 mmol/L), 50th (1.28 mmol/L), and 75th (1.54 mmol/L) percentiles. The follow up person-years, hazard ratios (HRs), and 95% confidence intervals (CIs) were computed for each category. The full model included age, race-ethnicity, education levels, physical activity, smoking status, alcohol use, BMI, systolic blood pressure, serum insulin level, serum triglyceride level, serum C-reactive protein level, serum creatinine level, and serum cholesterol level. For women, we also adjusted for menopausal status. All analyses were performed using SAS for Windows (version 9.2; SAS Institute Inc., Cary, North Carolina, USA).

## Results

The baseline characteristics of the study participants are shown in Table 1. The mean age was 46.2 years for men and 45.5 years for women. Approximately 77% of the participants were non-Hispanic whites. More than 40% reported they completed at least 13 years of education. Based on gender, 25.7% of men and 19.3% of women were current smokers, and 69.6% of men and 46.1% of women were identified as drinkers. A total of 79.3% reported engaging in regular exercise or physical activity. The mean serum C-peptide level was not significantly different between men and women. The mean serum HDL-C level was 1.2 mmol/L in men and 1.4 mmol/L in women.

**Table 1:** Baseline characteristics of study participants by sex from National Health and Nutrition Examination Survey III.

	Men		Women	
Variable	(n = 6,228)		(n = 6,957)	
Characteristic variables (N, Weighted %)				
Age groups (yr)				
20–29	1504	(25.4)	1683	(23.7)
30–39	1304	(26.9)	1611	(25.1)
40–49	1012	(19.8)	1124	(19.4)
50–59	618	(11.6)	746	(11.8)
60–69	814	(9.2)	726	(9.9)
70–79	547	(5.3)	584	(6.7)
80+	429	(1.8)	483	(3.3)
Race-ethnicity				
Non-Hispanic white	2553	(77.3)	2985	(77.1)
Non-Hispanic black	1607	(9.4)	1912	(10.7)
Mexican-American	1837	(5.7)	1759	(4.6)
Other	231	(7.6)	301	(7.6)
Education (yr)				
0–8	1520	(10.2)	1345	(9.8)
9–11	1038	(13.9)	1096	(11.7)
12	1727	(30.6)	2386	(37.0)
13+	1895	(45.3)	2091	(41.5)
Physical activity				
Yes	4803	(84.5)	4472	(74.4)
No	1425	(15.5)	2485	(25.6)
Smoking status				
Never	2162	(34.2)	4215	(54.5)
Former	2357	(40.1)	1626	(26.2)
Current	1709	(25.7)	1116	(19.3)
Alcohol use				
Yes	3866	(69.6)	2386	(46.1)
No	2188	(30.4)	4405	(53.9)
Measured Variables (Mean, SD)				
Body mass index (kg/cm^2^)	26.3	(4.7)	27.0	(6.3)
Systolic blood pressure (mmHg)	126.8	(17.2)	121.4	(20.5)
Diastolic blood pressure (mmHg)	76.5	(10.5)	71.9	(10.2)
Serum C-peptide (nmol/L)	0.7	(0.5)	0.7	(0.5)
Serum HDL cholesterol (mmol/L)	1.2	(0.4)	1.4	(0.4)
Serum insulin (pmol/L)	62.8	(49.1)	66.0	(52.0)
Serum triglycerides (mmol/L)	1.6	(1.1)	1.4	(0.9)
Serum cholesterol (mmol/L)	5.3	(1.1)	5.4	(1.2)
Serum C-reactive protein (mg/dL)	0.4	(0.7)	0.5	(0.8)
Serum creatinine (umol/L)	105.4	(25.2)	85.1	(22.2)

**Figure 1:**
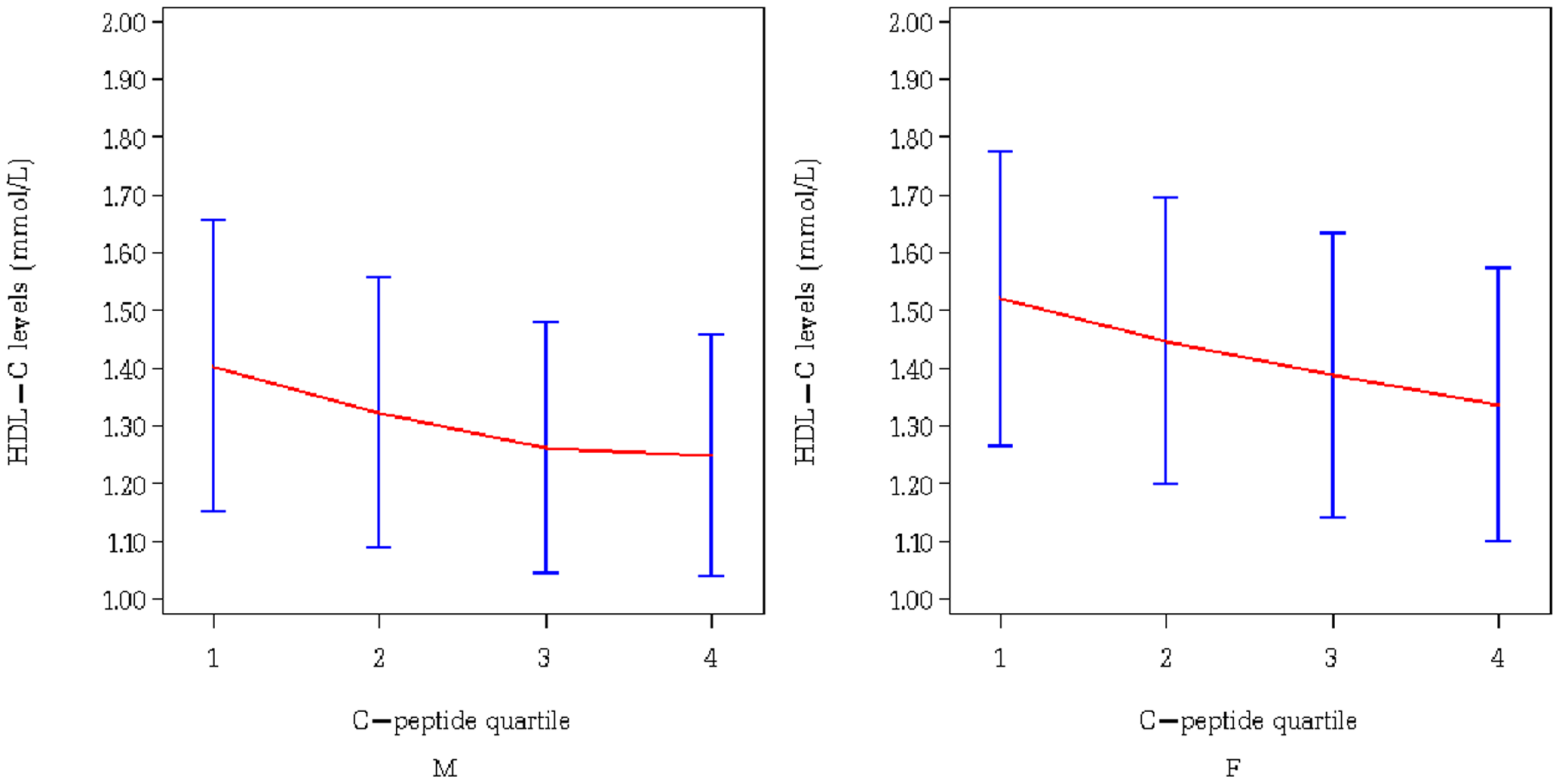
The mean serum HDL-C levels in different quartiles of serum C-peptide levels by sex.

Table 2 shows the differences in mean serum HDL-C levels between the quartiles of serum C-peptide is shown. Compared to the reference group (25th percentile), the 25–50th percentile group, 50–75th percentile group, and 75th percentile group had gradually lower serum HDL-C levels, which resulted in a significant linear trend. The mean serum HDL-C levels for the four quartiles of serum C-peptide levels by sex are illustrated in Figure 1.

**Table 2:** The mean and standard error for serum HDL-C levels in different quartiles of serum C-peptide levels by analysis of covariance.

	Levels of serum C-peptide (nmol/L)								
Variables	≤0.365		0.366–0.604		0.605–0.928		≥0.929		*p* for trend
	Mean	SE	Mean	SE	Mean	SE	Mean	SE	
Sex									
Men	1.42	(0.01)	1.26***	(0.01)	1.16***	(0.01)	1.07***	(0.01)	<0.001
Women	1.61	(0.01)	1.49***	(0.01)	1.37***	(0.01)	1.26***	(0.01)	<0.001
BMI (kg/cm^2^)									
<25	1.55	(0.01)	1.43***	(0.01)	1.35***	(0.01)	1.29**	(0.02)	<0.001
≥25	1.44	(0.01)	1.35***	(0.01)	1.25***	(0.01)	1.14***	(0.01)	<0.001
Insulin (pmol/L)									
<36.78	1.54	(0.01)	1.40***	(0.01)	1.30**	(0.03)	1.24	(0.07)	<0.001
36.78–74.95	1.48	(0.01)	1.39***	(0.01)	1.28***	(0.01)	1.21***	(0.01)	<0.001
>74.95	1.22	(0.07)	1.33	(0.03)	1.24**	(0.01)	1.16***	(0.01)	0.148

Covariates included in the analysis of covariance were age, race-ethnicity, education levels, physical activity, smoking status and alcohol use.

*** Comparisons significant at the <0.001 level.

** Comparisons significant at the <0.01 level.

*Comparisons significant at the <0.05 level.

HDL-C: high-density lipoprotein cholesterol.

The serum C-peptide level showed a strong negative association with serum HDL-C levels in the logistic regression analyses (Table 3). Compared with men with low serum C-peptide levels (≤0.365 nmol/L), the relative risk was 1.75 (95% CI, 1.50–2.05), 2.79 (95% CI, 2.36–3.31), and 3.07 (95% CI, 2.55–3.70) for men with serum C-peptide levels of 0.366–0.604 nmol/L, 0.605–0.928 nmol/L, and ≥0.929 nmol/L, respectively. The relative risk was 1.60 (1.36–1.89), 2.61 (2.22–3.09), and 3.67 (3.08–4.37) for women. In both men and women, the linear trend was significant (*p*<0.001).

**Table 3:** The odds ratios of quartiles of serum C-peptide levels for low serum HDL-C levels.

Serum C-peptide (nmol/L)	Odds ratio	95% confidence interval		*P* Value
Men				
≤0.365	Reference			
0.366–0.604	1.75	1.50	2.05	<0.001
0.605–0.928	2.79	2.36	3.31	<0.001
≥0.929	3.07	2.55	3.70	<0.001
	*p* for trend <0.001			
Women				
≤0.365	Reference			
0.366–0.604	1.60	1.36	1.89	<0.001
0.605–0.928	2.61	2.22	3.09	<0.001
≥0.929	3.67	3.08	4.37	<0.001
	*p* for trend <0.001			

Adjusted for age, race-ethnicity, education levels, physical activity, smoking status, alcohol use, body mass index, systolic blood pressure, serum insulin level, serum triglycerides level, serum C-reactive protein, serum creatinine and serum cholesterol level.

HDL-C: high-density lipoprotein cholesterol.

The survival rates of the study cohort are shown in Table 4. During the follow-up period, 588 deaths were observed (178912.2 person-years). Low HDL-C levels were negatively associated with CVD-related deaths. The HRs associated with reduced serum HDL-C levels for CVD-related deaths refer to the highest serum HDL-C levels (>75th percentile). In women, the HRs were 1.52 (95% CI, 1.05–2.20), 1.62 (95% CI, 1.10–2.53) and 1.60 (95% CI, 1.02–2.53) for HDL-C levels in the 50–75th percentile (1.29–1.54 mmol/L), 25–50th percentile (1.04–1.28 mmol/L) and 25th percentile (≤1.03 mmol/L). In men, the HRs were 1.48 (95% CI, 1.01–2.18) for HDL-C levels in the 25th percentile.

**Table 4:** The hazard ratios of low serum HDL-C levels for CVD events by sex in national health and nutrition examination survey III.

		CVD			
Serum HDL-C (mmol/L)	N	Events, n	Person-Years	HR (95% CI)	*p* Value
Men					
≥1.55	995	44	13551.8	Reference	
1.29–1.54	1257	50	17668.0	0.90 (0.58–1.39)	0.621
1.04–1.28	1682	100	24082.0	1.19 (0.81–1.74)	0.388
≤1.03	1939	161	27915.9	1.48 (1.01–2.18)	<0.044
Women					
≥1.55	2341	69	33048.8	Reference	
1.29–1.54	1850	64	26627.7	1.52 (1.05–2.20)	0.026
1.04–1.28	1565	59	22270.7	1.62 (1.10–2.53)	0.014
≤1.03	968	41	13747.4	1.60 (1.02–2.53)	0.041

Adjusted for age, race-ethnicity, education levels, physical activity, smoking status, alcohol use, body mass index, systolic blood pressure, serum insulin level, serum triglycerides level, serum C-reactive protein, serum creatinine and serum cholesterol level.

HDL-C: high-density lipoprotein cholesterol.

CVD: cardiovascular disease.

HR: hazard ratio.

CI: confidence interval.

## Discussion

This large, national population-based study demonstrated a strong negative dose–response relationship for serum C-peptide as a risk factor of CVD deaths in nondiabetic individuals with lower HDL-C levels. The observed association remained strong after adjustment for a number of potential confounders and stratified analyses for important risk factors. Moreover, the negative association presented a significant linear trend.

Recently, serum C-peptide as an important risk factor for CVD events or overall death has received increased attention. However, the underlying mechanism remains unclear. Evidence based on clinical, epidemiological, and experimental studies has shown that hyperinsulinemia is associated with a cluster of CVD risk factors, such as hypertension, obesity, elevated triglyceride levels, and decreased HDL-C levels [25]. It may also promote atherogenesis through its effects on lipid metabolism and blood pressure. Of the risk factors, obesity is one of the more important factors that may be associated with hyperinsulinemia [26]. However, a recent study found that serum C-peptide levels were more strongly associated with total and body regional fat distribution in nondiabetic subjects than was serum insulin levels [27]. Although several studies have shown that serum insulin was negatively associated with HDL-C levels and strongly associated with ischemic heart disease, the effect of serum C-peptide levels on HDL-C concentration is unclear. To the best of our knowledge, only one recent study has shown that those with high C-peptide levels were more likely to have a low HDL-C level in baseline observations [28]. In the present study, we identified a strong negative association between serum C-peptide levels and HDL-C levels; after adjustment for serum insulin level and potential confounding factors, the ORs of the three higher quartiles of serum C-peptide levels were significantly higher than the ORs of the three higher quartiles of serum insulin levels at 1.44, 2.37, and 3.06 in men and 1.34, 2.02, and 2.95 in women. In addition, the LURIC study demonstrated an association between serum C-peptide levels and cardiovascular mortality, and the results did not change after adjusting for HDL-cholesterol levels. However, the study participants were older-age Caucasian patients undergoing coronary angiography. These results suggest that, although the correlation between serum insulin and serum C-peptide is reported to be 60–70% [29], serum C-peptide also has potentially important physiological effects independent of serum insulin.

It has been well established that serum HDL-C is a significant and independent risk factor for CVD [30]. Therefore, it is an important and widely prevalent risk factor that has be addressed in the clinical context of primordial, primary, and secondary prevention. Attempts to increase HDL-C levels are becoming increasingly frequent in the literature, and most agree that increasing HDL-C levels should be one of the goals in the management of CVD risk reduction. The US National Cholesterol Education Program has recommended a therapeutic change in lifestyle behaviours, such as diet and physical activity. Additionally, in recent years, both basic and clinical studies have provided data suggesting that increased HDL-C levels may augment risk reduction in patients with CVD [31]. A number of therapeutic agents have been developed in an attempt to modulate serum HDL-C levels. However, a prospective review of 14 randomized trials was performed by the Cholesterol Treatment Trialists' Collaboration that included 90,056 subjects; after a 5-year follow-up, statin treatment did not appear to reduce the CVD risk associated with low HDL-C level [32]. Therefore, the causes and mechanisms of low HDL-C levels require further study; it is important to understand the possible mechanisms in order to target increased serum HDL-C levels for a reduced CVD risk.

Recent studies have also shown that low HDL-C levels are an independent risk factor for endothelial dysfunction and ultimately lead to CVD [33]. A balance between superoxide and nitric oxide (NO) release plays an important role in the maintenance of normal endothelial function [34], [35]. There is recent and increasing evidence that C-peptide might have a biological function in the physiology of microvascular blood flow regulation. Studies suggest that C-peptide administration to patients with type 1 diabetes results in concentration-dependent increases in blood flow in several tissues, improving the endothelial dysfunction; this action may be mediated in part by a NO-sensitive vascular mechanism [36], [37]. Studies have also shown that C-peptide significantly enhances the release of NO from bovine aortic endothelial cells; the effects are dose-dependent [38]. On the other hand, NO over-production may be caused by various diseases and can lead to severe health problems. Studies found that superoxide anions (O^2−^) and excess NO combine to form peroxynitrite (ONOO^−^). Under physiological conditions, ONOO^−^ can rapidly react with protein tyrosine residues or free tyrosine; nitration of free tyrosine and the protein tyrosine residues result in production of nitrotyrosine (NT). NT can induce nitration of HDL-C and produce a large number of oxidized HDL-C. Oxidized HDL-C may induce production of inflammatory cytokines and increase the risk of CVD. We assume that excessive C-peptide stimulation can cause dose-dependent increases in endothelial cells for the amount of NO released. HDL-C induces anti-oxidative effects and produces oxidized HDL-C. However, prospective basic and clinical experiments are required to validate the postulated mechanism. The present study indicates that serum C-peptide levels that are associated with CVD death in nondiabetic individuals can be indirectly caused, at least in part, by low serum HDL-C levels.

The use of a population-based cohort in the present study is a major strength; furthermore, data were collected through a large national surveillance system, and the analysis was enabled by highly representative biomarker data. We controlled for several important confounding factors, such as sex, BMI, and serum insulin levels using stratified analysis. In addition, to the best of our knowledge, this is the first study to explore serum C-peptide levels as an independent risk factor for CVD-related death in individuals without diabetes and its association with low serum HDL-C levels. ANCOVA and multiple logistic regression analyses were conducted to identify the strong association between serum C-peptide levels and low serum HDL-C levels and to obtain a linear trend after adjustment for a number of confounding factors. The study has also a major limitation; although this is a population-based cohort study, we only obtained cross-sectional data for biochemical measurements. We can verify the associations between serum C-peptide and serum HDL-C levels, but cannot determine causality. In addition, the baseline diagnosis of heart disease was based on a self-report questionnaire and the diagnosis itself was not available to identify cardiovascular events; therefore, there is a potential information bias.

In conclusion, previous studies have shown that serum C-peptide is an important risk factor for CVD events and related mortality. The results of the present study indicate that serum C-peptide levels are strongly and negatively associated with serum HDL-C levels and verify that low HDL-C levels can directly increase the risk of CVD death in individuals without diabetes in the general population. The association between serum C-peptide levels and serum HDL-C levels was independent of the serum insulin levels and resulted in a significant linear trend. Our results suggest that serum C-peptide may increase the risk of CVD events via a pathway that reduces HDL-C levels. However, further to explore the molecular and developmental mechanisms underlying have important clinical and public health implications.
